# Impact of COVID-19 on adolescent sexual life and attitudes: have we considered all the possible secondary effects of the pandemic?

**DOI:** 10.1007/s00431-023-04878-5

**Published:** 2023-03-20

**Authors:** Eirini Kostopoulou

**Affiliations:** Department of Paediatrics, University General Hospital of Patras, University of Patras, Patras, 26504 Greece

**Keywords:** Pornography use, Adolescents, COVID-19, Aggressiveness, Sexual behaviour

## Abstract

A significant increase in pornography use has been reported in the adolescent population worldwide over the past few years, with intensification of the phenomenon during the COVID-19 pandemic. The aim of the present review is to provide data on the frequency of pornography consumption among adolescents during the pandemic and raise awareness about its potential impact on personal beliefs and sexual attitudes in the long term. A comprehensive literature review was performed in two scientific databases using the crossmatch of the terms “pornography”, “adolescents” and “COVID-19”. A significant increase in pornography consumption in adolescents was documented during the COVID-19 pandemic as a result of social detachment. Fulfilment of sexual desires in the context of social distancing, alleviation of COVID-19-related boredom and psychological strain, and coping with negative emotions are some of the reported reasons for increased pornography use during the pandemic. However, concerns have been raised in the literature regarding potentially negative effects of excessive pornography use from an early age, including the development of pornography addiction, sexual dissatisfaction and aggressive sexual attitudes reinforced by gender preoccupations and sexual inequality beliefs.

*Conclusion*: The extent to which increased pornography consumption from an early age during the COVID-19 pandemic may have affected adolescents’ mental well-being, personality construction and sexual behaviour is yet to be seen. Vigilance from the society as a whole is required so that potential negative adverse effects of adolescent pornography use and potential social implications are recognized early and managed. Further research is needed so that the full impact of the COVID-19-related pornography use in the adolescent population is revealed.

**Table:** 

**What is Known:** *•A significant increase in pornography consumption has been documented in the adolescent population worldwide over the past decades due to its quick, affordable and easy access from electronic devices and the possibility of anonymous and private participation.* *•During the COVID-19 pandemic, this phenomenon was intensified as a coping mechanism to social isolation and increased psychosocial strain.*	
**What is New:** *•Concerns have been raised regarding the risk of pornography addiction in adolescents during the COVID-19 pandemic, making the post-pandemic adaptation challenging.* *•Awareness is raised in parents, health care providers and policy makers about the potential negative impacts of pornography consumption from an early, vulnerable age, such as sexual dissatisfaction and development of aggressive sexual attitudes and sex inequality beliefs.*

**What is Known:**

*•A significant increase in pornography consumption has been documented in the adolescent population worldwide over the past decades due to its quick, affordable and easy access from electronic devices and the possibility of anonymous and private participation.*

*•During the COVID-19 pandemic, this phenomenon was intensified as a coping mechanism to social isolation and increased psychosocial strain.*

**What is New:**

*•Concerns have been raised regarding the risk of pornography addiction in adolescents during the COVID-19 pandemic, making the post-pandemic adaptation challenging.*

*•Awareness is raised in parents, health care providers and policy makers about the potential negative impacts of pornography consumption from an early, vulnerable age, such as sexual dissatisfaction and development of aggressive sexual attitudes and sex inequality beliefs.*

## Introduction

Over the past decades, western societies have experienced a revolution in internet communication technology and massive access to online information from an early age. Since internet access became widely accessible, it is well known that a significant percentage of the adolescent population lives digital lives and that internet represents a central feature of their identity. Among the information consumed through digital media, online pornographic material is also included [1].

Indeed, it has been widely described that pornography use is on the rise in adolescents [2], which requires attention as this is a critical period of sexual development [3–7]. Availability of mobile internet from earlier ages has removed previous barriers to accessing pornography and is synchronous to the social acceptability of pornography use [2].

During the course of the COVID-19 pandemic, this phenomenon became more intense as a result of social detachment. An unprecedented increase in pornography consumption was documented worldwide both in adults and in adolescents [8–13], also confirmed by a surge in traffic on pornographic websites reported after the outbreak of the pandemic [14, 15]. The physical distancing policies that were imposed in order to minimize the risk of transmission of the virus translated into social isolation and increased psychosocial strain worldwide. These measures are believed to have changed social, interpersonal and potentially sexual relationships in adults [16]. It has been proposed that high-frequency pornography consumption may require clinical attention due to potential psychological and physical negative outcomes in adults [17]. Little is known about how the pandemic has affected adolescents’ sexual life. Studies are scarce and limited; however, concerns have been raised in the literature regarding potential harms of the pandemic-related pornography consumption on adolescents’ interpersonal relationships, but also on their sexual beliefs and attitudes in the long term [3, 18, 19].

The aim of the current review is to provide existing data on adolescent pornography use during the COVID-19 pandemic, taking into consideration aspects such as frequency of consumption and the potential impact of this recent phenomenon on adolescent sexual life and behaviour in the long term.

## Methods

**Fig. 1:**
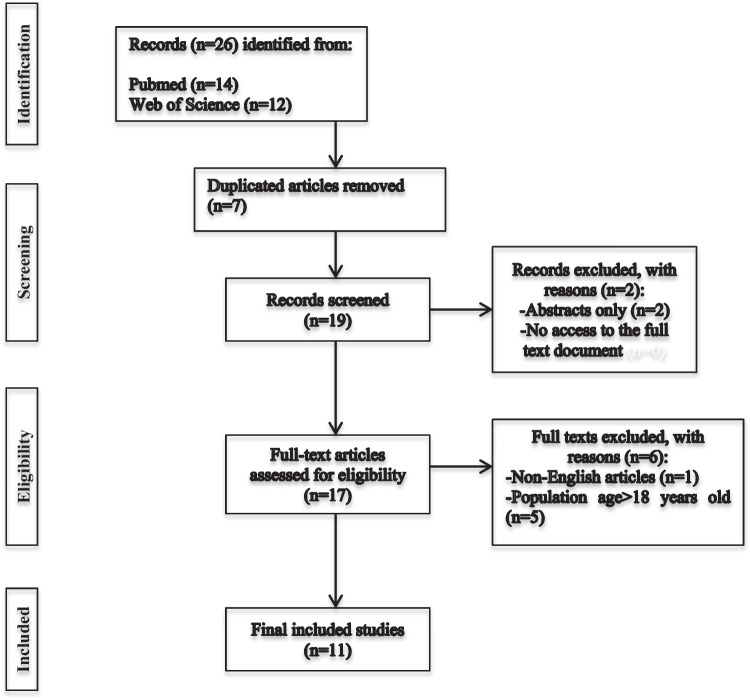
PRISMA flow diagram describing the stages of the search strategy

A comprehensive literature research on adolescent pornography use and adolescent pornography use during the COVID-19 pandemic was performed. Data were extracted from two different scientific databases, Medline (PubMed) and Web of Science, to include studies up to 2023. The search terms included “pornography”, “adolescents” and “COVID-19”. The Preferred Reporting Items for Systematic Reviews and Meta-Analyses (PRISMA) guideline and checklist was used for the conduction of this review (Fig. 1).

### Eligibility criteria

The eligibility of the studies was assessed in two steps. First, the selection of studies was based on the relevancy of titles and abstracts. In the second step, the relevancy of the full texts of the remaining studies was assessed based on the eligibility criteria of the present study.

The inclusion criteria for the articles selected were as follows: (a) being published in peer-reviewed journals; (b) referring to pornography use during the COVID-19 pandemic, (c) being published after 2020, (d) referring to adolescents < 18 years old, (e) being written in English. Exclusion criteria were applied for (a) abstracts or conference abstracts without published full text and (b) articles written in other than the English language.

### Data extraction

The information extracted from full texts of the selected articles included the first author name, country of study, year of publication, study design, sample characteristics, existing knowledge on pornography use in adolescents and the possible role of the COVID-19 pandemic on pornography use in adolescents.

### Selection process

A total of 26 articles were collected during the initial search. After duplicate removal, 7 publications were excluded. After the eligibility criteria were applied, and based on the relevancy of the title and the abstract and following the full-text screening, 11 articles were finally included (Table 1).

**Table 1:** Summary of included articles

**Author(s), publication year**	**Aim**	**Country**	**Study design**	**Sample characteristics (sample size, age)**	**Period of study**
Petrovic et al., 2022 [49]	To describe the internet use and internet-based addictive behaviours	Croatia	Narrative review	N/A	2019–2022
Bőthe et al., 2022 [48]	To document potential changes in adolescents' pornography use frequency, motivations and problematic pornography use before and during the COVID-19 pandemic	Canada	Longitudinal study	*N* = 1771, 15.42 ± 0.59	Pre- and post-COVID-19
Maes and Vandenbosch, 2022 [85]	To explore adolescents’ changes in the frequency of sexually explicit internet material use before, during and after a strict lockdown period in Belgium	Belgium	Three-wave longitudinal study	*N* = 522, 15.39 ± 1.42	Pre-, during and post-strict lockdown period
Awan et al., 2021 [12]	To show the rise in pornography use during lockdowns in different countries worldwide	Pakistan, USA, Nigeria, Italy, New Zealand, India	Review	N/A	Lockdown periods
Masaeli and Farhadi, 2021 [57]	To investigate the possible increase in Internet-based addictive behaviours during the COVID-19 pandemic	Iran	Systematic review	N/A	2020
Nelson et al., 2020 [23]	To explore how the COVID-19 pandemic and physical distancing measures have impacted the well-being and sexual health among adolescent sexual minority males (ASMM) during the initial phase of physical distancing mandates in the USA	USA	Cross-sectional survey study	*N* = 151, 14–17 years old	March 2020–May 2020
Perissini et al., 2020 [86]	To comment on the increase in pornography use and early development of beliefs and sexual attitudes in adolescents during the initial phase of the COVID-19 pandemic	Brasil	Editorial	N/A	Initial phase of COVID-19 pandemic (2020)
Kiraly et al., 2020 [91]	To describe the development of problematic internet usage patterns during the COVID-19 pandemic and make practical recommendations to help diminish them	Spain, USA, Japan, Iran, Germany, South Korea, South Africa, Lithuania, UK, Hungary, Switzerland, Italy, New Zealand, North Macedonia, Israel, Australia, Canada	Consensus guidance	N/A	Initial phase of COVID-19 pandemic (2020)
Socias et al., 2020 [92]	To describe the main family prevention measures applied in Spain against family violence and the abusive consumption of gambling or pornography by children and adolescents during the COVID-19 period	Spain	Review	N/A	Initial phase of COVID-19 pandemic (2020)
Lukavska et al., 2021 [93]	To assess the perceived importance and extent of school-based preventions related to screen-related risk behaviours during the long-term, nation-wide distant schooling period in the Czech Republic in children and adolescents	Czech Republic	Online survey	*N* = 1698, School based prevention specialists	Distant schooling period
Scull et al., 2022 [94]	To assess the immediate (post-test) and short-term (3-month) effects of Media Aware, a web-based comprehensive sexual health program, on adolescents’ media, sexual health and communication outcomes during the COVID-19	USA	Randomized controlled trial	*N* = 590, 9th–10th grade students	2019–2020

## Results

### Motivations for pornography use

Seeking for sexual pleasure is the most common reason for pornography use in adults and adolescents [4, 20]. Due to its accessible, affordable and anonymous nature, pornography consumption enables the fulfilment of sexual desires particularly when the possibility of casual sex is limited, as in the presence of social distancing requirements [21]. Indeed, during the restrictive and containment measures imposed by the COVID-19 pandemic, pornography use and autoerotism became channels through which sexuality was expressed. This unprecedented consumption of online pornography during the pandemic compared to the pre-pandemic era was not only fuelled by social isolation, but hypothetically, also caused by it [15]. Alternatively, the increased time spent at home during the lockdowns was associated with increased engagement in internet use and screen-based activities, including pornography use [22–24].

Although not all studies have demonstrated that social distancing during the pandemic resulted in less sexual interaction among adults [25], a significant number of studies from different countries have shown a decrease in partnered sex and increased online dating during the pandemic [26–29]. Similar results were demonstrated in studies on adolescents and young adults, who turned to digital forms of communication with partners and to digital pornography use during the pandemic [23, 30, 31].

Other reasons of pornography use include sexual curiosity, self-exploration and the need for stress reduction, alleviation of boredom and coping with negative emotions. In this regard, pornography use in adults has been described as a “constructive coping behaviour” to overcome “boredom and fear” caused by the pandemic, also known as “eroticization of fear” [32]. In contrast, for some authors, increased pornography use could represent a maladaptive coping strategy and dysfunctional behaviour toward stressors, such as loneliness, fear and feeling of powerlessness triggered by situations such as the pandemic [33, 34]. Previous studies have shown that adolescents, who seem to have suffered greatly from psychological consequences during the COVID-19 pandemic, use pornography to cope with negative feelings [35, 36].

Another particularly interesting approach of pornography use is that of “escapism”. It has been proposed that online pornography offers the individual the possibility to escape from the real world and participate in an artificial and more perfect sexual situation, with an “ideal self”. This can be liberating for individuals suffering from social anxiety or body image issues, as sexual reward can be achieved in privacy and through body image avoidance [37, 38].

### Frequency

Increased frequency of pornography use in male and female adolescents has been documented worldwide, partly due to peer influence and the need for autonomy. According to large-scale, nationally representative data from the USA, Canada and Europe, 63–68% of adolescents reported lifetime pornography use [39, 40]. Although girls and women consume online pornography, boys and men consume pornography at higher rates [13, 18, 41–47]. In a study by Bőthe et al., boys reported not only more frequent pornography use, but also higher levels of problematic pornography use than girls. However, the majority of adolescents engaging in pornography use reported no negative impact on their life or distress [48].

In addition to the increasing frequency of pornography use, the age of first pornography use seems to also be declining [2, 41]. Interestingly, little parental supervision and dysfunctional or weak family relations have been associated with increased pornography use during adolescence [6].

### Concerns regarding pornography use in adolescents during the COVID-19 pandemic

(i) Addiction

The increasing rates of pornography use among adolescents over the past few years, showing a surge during the COVID-19 pandemic, have raised concerns with regard to the development of addiction due to the vulnerability of the adolescent developing brain [49]. According to a longitudinal study on internet addiction, among the internet-related activities, online pornography was the most likely to be addictive [50]. “Internet pornography addiction” or “problematic online pornography use”, an under-researched phenomenon, is placed under the umbrella construct of hypersexual or “compulsive sexual behaviour”. Rousseau et al. have demonstrated a positive association of depressive and anxiety symptoms with problematic pornography use in adolescents in a recent longitudinal study [51]. Problematic pornography use has been identified in 5–14% of adolescents [52, 53].

In adults, it has been proposed that problematic internet use may be the result of the combination between an underlying vulnerability with a stress factor, such as the COVID-19 pandemic [54–56]. Also, that internet addiction could in the long term have a negative impact on psychosocial and physical well-being [57].

Addictive behaviours, such as internet gaming or pornography, have been associated with dysfunctions in dopaminergic circuits that feed reward mechanisms [58]. In a study published in 1951, it was reported that a greater activation of the brain’s reward systems occurs by an artificial stimulus compared to a natural stimulus of a similar type [59]. In 2010, Barrett described internet pornography as an example of supranormal stimulus, due to the numerous artificial scenarios available to the consumer to choose from, seeking for new, more perfect content, thus greater reward, entering the “addictive mode” [60]. Also, similarities have been found in brain dysfunction between substance and pornography addiction [61].

In the pandemic context, adoption of a particular lifestyle for elongated periods can make post-pandemic “re-adaptation” difficult and engagement in physical sexual activity dysfunctional [62].

(ii) Sexual dissatisfaction/frustration

Pornography may affect different aspects of sexual life, including sexual satisfaction [63]. Long-term pornography use is associated with disappointment and reduced sexual satisfaction due to the supranormal nature of pornography and unrealistic expectations of sexual performance in real life [64], although it is not considered a major risk factor of sexual dysfunction [65]. Adolescents may perceive pornography as real due to lack of real-life experiences [27]. As a result, personal insecurities about adolescents’ bodies, physical appearance and sexual performance may develop [66]. A recent expression of pornography-related frustration regarding body image dissatisfaction involves decisions concerning genital cosmetic surgery in females have been associated with pornography use and, specifically, with negative genital self-image despite the genitalia being in a normal range or with negative comments from sexual partners [67–69].

In addition, it has been suggested that the distance between reality and fantasy may cause reduction in desire and interest in sex [70]. More importantly, pornography use has been associated with impaired mental well-being reflected by lower self-esteem, life satisfaction and increased depression symptoms among adolescents [6]. This is particularly worrying considering the elevated stress levels and increased mental health issues reported during the pandemic in children and adolescents [71, 72]. However, a causal relationship between pornography and mental well-being constructs has not been established with certainty [73].

Furthermore, excessive pornography consumption may affect the quality of intimate relationships [74]. Specifically, it has been inversely associated with relationship satisfaction and closeness and positively associated with depression and loneliness, which may also explain the documented increase in pornography use during the pandemic. Although solitary sexual desire may be satisfied through fake virtual relationships, the need for affection, intimate relationships and dyadic sexual desire cannot be fulfilled by this means [8].

(iii) Shaping of aggressive and sex inequality attitudes

The possible impact on adolescents’ sexual attitude and belief system constitutes one of the most worrying aspects of pornography use during this vulnerable age. Children and adolescents are considered the most vulnerable audiences to sexually explicit material [5]. Due to reduced control over impulses, adolescents have the propensity to mimic sexual behaviours and their sexual attitude is more likely to be defined by pornographic scenes they have viewed. The more frequently someone consumes pornography, the more the person’s sexual behaviour is guided by the viewed scenes [75, 76]. Such scenes often include aggressiveness and images of abuse favouring gender stereotypes and gender-inequality beliefs [3, 6, 62, 75, 77]. As a result, expectations about what behaviours partners should engage in are developed and reinforced [2, 42].

Worryingly, several studies have reported significant relationship between online pornography consumption and attitudes supportive of violence and tolerance of sexual violence toward women [42, 43, 62, 78, 79]. A study that examined 172 online pornography videos demonstrated that male performers caused intentional harm and pain in female performers, whereas 58% of the videos showed non-consensual sexual interactions [21]. Another study showed that exposure to online pornography at an early age is associated with increased risk of juvenile sexual perpetration [21, 44, 47, 63]. Furthermore, according to an anonymous survey among 4564 teenagers aged 14–17 years old, male teenagers who were frequent consumers of online pornography were more likely to admit sexual coercion or using force to obtain sexual intercourse [44]. Exposure to sexual images of pleasure on sexually violent acts, where women’s consent is not necessary, or of male domination over females and tolerance by female performers who react positively to humiliation and violence, may promote a culture of tolerance to sexual behaviours that reinforce sexual inequality and non-consensual intercourse [80, 81].

In the same context, criticism has been raised regarding the objectification of women, who are transformed into sexual artifacts and dehumanized to the point where their role is to provide sexual pleasure to men [82]. Girls report being pressured by their boyfriends into watching pornography and re-enacting pornographic scenes watched [83]. They also might get confused about what their role is in a sexual relationship, and may engage in behaviours that they are not comfortable with or that are against their will, either because they are forced by their partners or because they perceive that they have the obligation to do so.

In addition, adolescents have limited ability to differentiate between what is”normal” and “not normal” sexual behaviour, thus pornography consumption is thought to encourage eccentric practices and social norm rule breaking [6]. Frequent consumers may potentially become desensitized to sexual behaviours that have previously been believed to be unusual [42].

### Miseducation

Another aspect to be considered is the replacement of the conventional ways of sexual education by pornography use. Learning about sex through distorted versions of masculinity and sexual behaviour, in an uncontrolled and non-scientific context, may influence sexual beliefs and have a negative impact on adolescents’ sexuality [66, 70, 84–86].

## Conclusion

Pornography consumption in adults and adolescents has risen to a worldwide proportion due to quick and easy access from electronic devices and the possibility of anonymous and private participation. During the COVID-19 pandemic, this phenomenon became more intense due to its association with physical contact [87, 88]. In addition, the psychosocial burden imposed by the pandemic, the emergence or deterioration of mental health issues in the adolescent population and the risk of addictions further contributed to the intensification of pornography use as a coping mechanism, making post-pandemic “re-adaption” challenging [11, 71, 89].

The current review summarizes viewpoints on the possible impact of pornography use during the COVID-19 pandemic on sexual life of adolescents, a meaningful aspect of health development. It highlights potential immediate and long-term future adverse effects of pornography addiction in this population, an underestimated contemporary issue that requires attention. Questions arise as to what the role of pornography use is in the development of personal preoccupations and attitudes that might determine future sexual behaviour and intimate relationships, the construction of personality, and the perception of equality between the sexes. Also, concerns about what the appropriate lower age, if any, for pornography use is, so that negative personal and social implications are prevented.

The presented data bring to surface concerns regarding the absence of sex education before and after the pandemic in many parts of the world. This, in association with insufficient parental supervision and reluctance to engage children and adolescents in discussions about their sexual life or pornography consumption, results in learning for sex through inappropriate sources. Thus, the present work aims to raise awareness regarding potential negative impacts of pornography use and the need for prevention education and early intervention, particularly during and after emergencies, such as the COVID-19 pandemic.

Parents should be made aware of the importance of their involvement so that potential harms of uncontrolled pornography use, such as its addictive potential during adolescence and the possibility of development of gender stereotypes, are prevented [2, 41, 90].

Furthermore, prevention efforts, including educational programs, need to be implemented in the school and other settings to educate where porn misinforms. Sexual education strategies should be directed toward helping children have more safe and positive online experiences and lead respectful, coercion-free sexual relationships. Health care professionals also need to be educated on diagnostic tools for detecting problematic pornography use and on appropriate intervention methods [1, 2, 47, 91].

It becomes apparent that cooperation between all parts involved, including parents, schools, health care providers, social services and policy makers, is of major importance. Against influences favouring aggression, sex inequality, misogynistic, offending and humiliating behaviours, vigilance from society as a whole is warranted and implementation of education strategies with an emphasis to moral values and principles based on equality and respect.

Would it be an exaggeration to assume that excessive pornography consumption from an early age is a risk factor for problematic sexual behaviour and a step backwards to the victory of equality? Have we considered all the secondary impacts of COVID-19, including the full impact of the pandemic on adolescents’ sexuality? As this is an understudied field, further studies are needed before safe conclusions are drawn.

## Data Availability

The author confirms that the data supporting the findings of this study are available within the article [and/or] its supplementary materials.

## Abbreviations

COVID-19: Corona virus disease-19
